# Achilles tendon moment arm in humans is not affected by inversion/eversion of the foot: a short report

**DOI:** 10.1098/rsos.171358

**Published:** 2018-01-10

**Authors:** Susann Wolfram, Christopher I. Morse, Keith L. Winwood, Emma Hodson-Tole, Islay M. McEwan

**Affiliations:** 1 Department of Exercise and Sport Science, Health, Exercise and Active Living (HEAL) Research Centre, Manchester Metropolitan University, Crewe, UK; 2 School of Healthcare Science, Manchester Metropolitan University, Manchester, UK

**Keywords:** magnetic resonance imaging, centre of rotation, musculoskeletal modelling

## Abstract

The triceps surae primarily acts as plantarflexor of the ankle joint. However, the group also causes inversion and eversion at the subtalar joint. Despite this, the Achilles tendon moment arm is generally measured without considering the potential influence of inversion/eversion of the foot during plantarflexion. This study investigated the effect of foot inversion and eversion on the plantarflexion Achilles tendon moment arm. Achilles tendon moment arms were determined using the centre-of-rotation method in magnetic resonance images of the left ankle of 11 participants. The foot was positioned at 15° dorsiflexion, 0° or 15° plantarflexion using a Styrofoam wedge. In each of these positions, the foot was either 10° inverted, neutral or 10° everted using an additional Styrofoam wedge. Achilles tendon moment arm in neutral foot position was 47.93 ± 4.54 mm and did not differ significantly when the foot was positioned in 10° inversion and 10° eversion. Hence, inversion/eversion position of the foot may not considerably affect the length of the Achilles tendon moment arm. This information could be useful in musculoskeletal models of the human lower leg and foot and when estimating Achilles tendon forces during plantarflexion with the foot positioned in inversion or eversion.

## 1. Introduction

The Achilles tendon (AT) is the common tendon of the triceps surae muscle group consisting of soleus, gastrocnemius medialis and gastrocnemius lateralis. It inserts at the posterior aspect of the calcaneus and through force production of the triceps surae generates a plantarflexion moment at the talocrural joint. The magnitude of triceps surae muscle force that is converted into joint moment is determined by the moment arm of the AT. The moment arm is the perpendicular distance from the tendon to the joint centre of rotation (COR) and the COR represents one point along the rotational axis of the articulation between the bodies of the tibia and the talus. The AT moment arm is frequently determined in order to estimate musculo-tendon forces in musculoskeletal models, for example during running [1,2].

The length of the AT moment arm is not constant, but rather is dependent on the location and orientation of the talocrural joint axis in three-dimensional space. The talocrural joint axis location, in turn, is dependent on talocrural joint angle, and muscle activation level [3,4]. For example, in neutral talocrural joint position (foot perpendicular to the lower leg), Fath *et al*. reported an AT moment arm of 34.3 ± 4.5 mm calculated with the tendon excursion method, while the moment arm in the same position was longer at 51.7 ± 4.3 mm using the COR method [5]. With an increase in plantarflexion effort, the triceps surae bulges and pushes the AT away from the talocrural joint COR. Hence, the AT moment arm increases [3,5,6]. Furthermore, the moment arm of the AT decreases when the talocrural joint is dorsiflexed and increases when in a plantarflexed position [5,6]. This is due to increased AT curvature in a plantarflexed position [6] and posterior translation of the talocrural joint axis from a dorsiflexed to a plantarflexed position [7].

Differences in the reported moment arms are due to the underlying assumptions on which both methods of estimating the AT moment arm are based. The tendon excursion method relies on the principle of virtual work and the AT moment arm is estimated based on the ratio of the linear tendon displacement and the joint angle excursion. Knowledge of the location of the COR is not required. The COR method is a two-dimensional geometric method, with which the location of the talocrural joint COR is identified based on the underlying anatomy. The AT moment arm is then measured as perpendicular distance between the AT and the identified COR. Both methods correlate strongly with each other [5] but either under- or overestimate the length of the AT moment arm. The tendon excursion method underestimates the length of the AT moment arm because it is assumed that no internal forces are present in the joint of interest and that any tissue excursion is due to joint rotation, thereby neglecting the effect of slack within the tendon [8]. The COR method overestimates the length of the AT moment arm because it is assumed that the talocrural joint axis is located perpendicular to the sagittal plane but, in fact, the joint axis deviates from the sagittal plane by several degrees [7,9,10]. This could be overcome with recently emerging three-dimensional methods for the estimation of AT moment arms [9,11,12].

The effect of plantarflexion and dorsiflexion on the plantarflexion AT moment arm is well established, but the effect of inversion and eversion has not been studied extensively. Activation of gastrocnemius medialis not only causes plantarflexion of the talocrural joint but also inversion of the subtalar joint [13]. This suggests an interdependence of talocrural joint and subtalar joint movements, which may affect AT moment arm length. Furthermore, inversion is accompanied by plantarflexion and eversion by dorsiflexion [14]. Inversion and eversion movements of the subtalar joint have been shown to cause a change in the location and orientation of the rotational axis of the talocrural joint [15], but it is not known if the inversion/eversion position of the foot and the associated variation in talocrural joint axis position have an effect on the moment arm of the AT.

It is important to know whether AT moment arm changes occur when the inversion/eversion position of the foot is altered to determine mechanical properties of the AT. To determine AT mechanical properties during plantarflexion contractions, the AT moment arm must be determined as precisely as possible for an accurate calculation of tendon forces and stiffness. Inversion/eversion of the foot has, furthermore, been suggested to be a contributing factor to regional loading patterns within the triceps surae muscle group and the AT [16]. A recent *in vitro* study found that strain on the lateral side of the AT increased when the calcaneus is in an inverted position while strain is increased on the medial side of the AT when the calcaneus is in an everted position [17]. The purpose of this study was, therefore, to examine the effect of the inversion/eversion position of the foot on the moment arm of the AT in healthy adults without musculoskeletal pathologies. We hypothesize that the AT moment arm is greater in inversion and smaller in eversion in comparison to a neutral foot position.

## 2. Material and methods

### 2.1. Magnetic resonance image acquisition

Sagittal plane scans of the left rearfoot of 11 participants (six males, five females, age 28.1 ± 4.8 years, height 174.2 ± 9.3 cm, body mass 70.1 ± 11.2 kg) were obtained for AT moment arm analysis. Participants reported no musculoskeletal injury to their foot, ankle or lower leg within the previous 6 months. Each participant was asked to lie in the supine position on the bed of a 0.2 T magnetic resonance imaging (MRI) scanner (Esaote, Italy) with their left ankle positioned within the regional coil, ensuring that the entire free AT and the calcaneus were visible when scanned. A total of nine scans were conducted in a systematic order as described below. The following scan parameters were used for each scan: TR/TE/flip angle was set at 580 ms/16 ms/75°. Twelve slices with a thickness of 4 mm were obtained during each scan with a gap between slices of 0.4 mm resulting in a field of view of 52.8 mm. The duration of each scan was 1.55 min.

Styrofoam wedges were used to alter the participants' foot into plantarflexed/dorsiflexed and inverted/everted positions. Three scans of the participants' left rearfoot were taken with the foot in neutral position (neither inverted nor everted) in 15° plantarflexion, 0° and 15° dorsiflexion, respectively. The plantarflexed and dorsiflexed scans were conducted to determine the COR for the talocrural joint at 0° (foot perpendicular to the lower leg). The procedure was repeated for the foot in 10° inversion and 10° eversion, respectively. During the scans, the participants were asked to maintain good contact with the wedge throughout the scan and care was taken to align the foot vertically each time.

### 2.2. Determination of Achilles tendon moment arm

**Figure 1.**
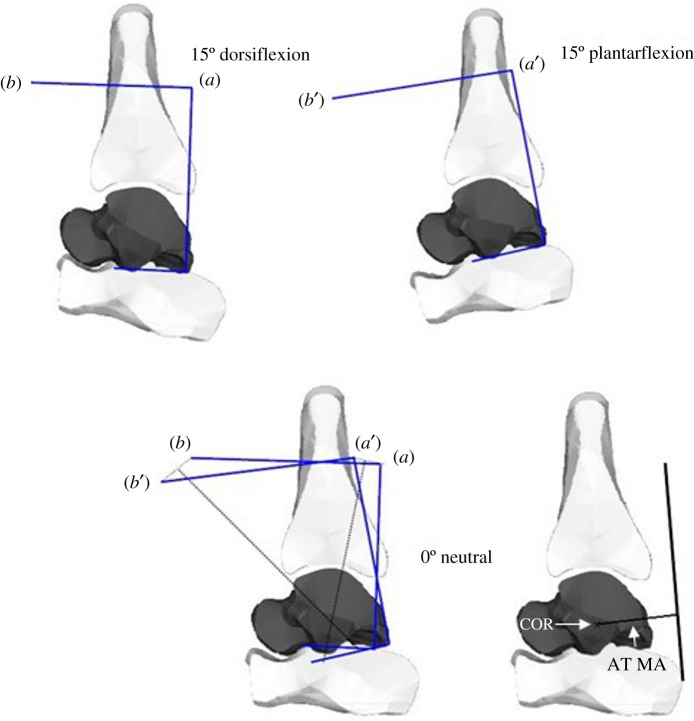
Schematic depiction of the COR method to determine the AT moment arm. Two anatomical landmarks are marked on the talus in dorsiflexed and plantarflexed position, respectively. From the straight line connecting them, two more straight lines are drawn at a perpendicular angle. The resulting figures are overlaid on the neutral position image. The intersection of the straight lines drawn from *a*–*a'* and *b*–*b'* represents the COR. The AT moment arm is the shortest distance between the tendon and the COR.

The resulting image series were analysed using the COR method, which is a two-dimensional geometric method that identifies a single joint COR along the joint axis of rotation. The position of the COR of the talocrural joint, the location of the AT (representing the location of the force vector) and thus the moment arm of the AT in the talocrural joint neutral position were identified using an open-source medical imaging software (OsiriX v. 5.8.8.5, Pixmeo, Bernex, Switzerland). Based on the description by Reuleaux, the tibia was assumed to be the non-moving part of the joint, with the talus rotating around it [18]. The procedure was described in detail previously [4,6] and is schematically depicted in figure 1. The AT moment arm was estimated as the perpendicular distance between the longitudinal axis of the AT and the COR. The COR and, therefore, the moment arm of the AT were determined for the foot in neutral, 10° inversion and 10° eversion positions.

### 2.3. Statistical analyses

Statistical analyses of the obtained data were performed in SPSS (v. 22.0, Chicago, IL). Values for AT moment arms are represented as mean ± s.d. A one-way ANOVA was used to test for differences in AT moment arms between the three foot positions. A Bonferroni *post hoc* test was performed when differences between groups were indicated. The level of significance was *α *= 0.05.

## 3. Results

**Figure 2.**
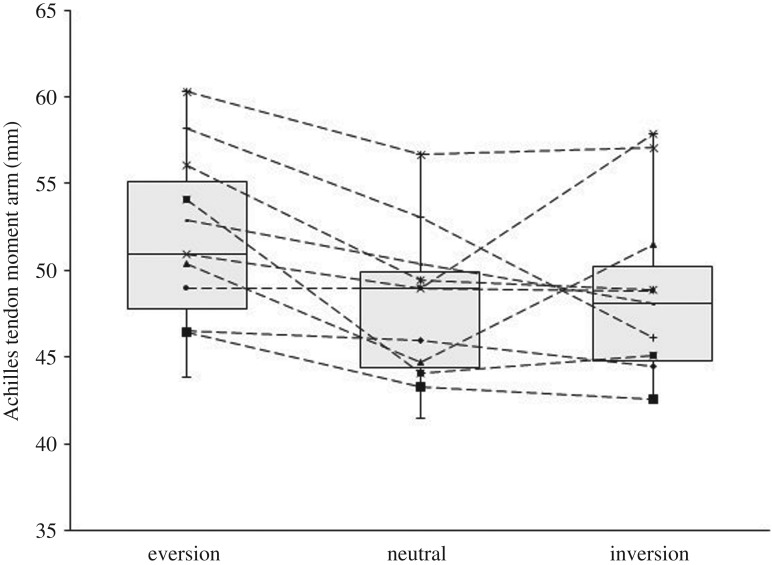
Box-and-whisker plot of AT moment arm in calcaneal neutral, inversion and eversion positions. One box shows the data distribution for one foot position as first, median and third quartile. Whiskers represent the variability of the data as 1.5 times the interquartile range. Line graphs represent individual participants. The differences in AT moment arm in everted, neutral and inverted positions were not statistically significant (*p* > 0.05, *n* = 12).

The AT moment arm in the neutral, inversion and eversion foot positions was 47.93 ± 4.54 mm, 48.6 ± 5.1 mm and 51.7 ± 5.2 mm, respectively. There was no significant change in AT moment length in both inversion and eversion positions of the foot (*p *= 0.823, figure 2).

## 4. Discussion

The present study investigated the relationship between the AT moment arm and inversion/eversion position of the foot in healthy, non-pathological adults. Previous studies have identified a change in AT moment arm with dorsiflexion and plantarflexion of the talocrural joint in this population, but the relationship of AT moment arm with inversion and eversion of the foot has not been previously investigated [4,6]. We hypothesized the AT moment arm to be greater in an inversion position of the foot and smaller in an eversion position of the foot compared to a neutral foot position. The results of this study, however, show that the AT moment arm estimated with the COR method is similar regardless of the inversion/eversion position of the foot indicating that the location of talocrural joint axis with respect to the AT is not dependent on calcaneal inversion and eversion. Therefore, the hypothesis must be rejected.

Inversion and eversion of the foot occur at the subtalar joint whose joint axis location and orientation are highly variable during these movements and between individuals [19–21]. Inversion and eversion movements of the foot also have an effect on the orientation and location of the rotational axis of the talocrural joint [15,22]. Lundberg *et al*. showed that inversion and eversion of the foot resulted in considerable changes of the angle between the talocrural joint axis and the sagittal plane and transverse plane, respectively [15]. However, the angle between the talocrural joint axis and the frontal plane remained nearly constant. Therefore, the distance between the talocrural joint axis and the AT can be assumed to be the same regardless of the inversion or eversion position of the foot. The axes of both subtalar joint and talocrural joint have been studied extensively and large variations in the location of these joint axes have been reported [10,15,23,24]. The relationship between subtalar and talocrural joint axis, however, has not been studied in great detail. Parr *et al*. used a three-dimensional vector angle between both joint axes to describe their relationship, but they did not find differences between different ethnical groups nor between males and females [25]. The results presented here provide further support for this. A difference in the AT moment arm in different inversion/eversion positions of the foot was not found suggesting that the location of the COR rotation of the talocrural joint estimated with the COR method does not shift in relation to the inversion/eversion position of the foot.

In the present study, MRI scans were taken in non-weight bearing, but with the foot in good contact with the wedge so that a closed kinematic chain situation can be assumed. In this situation, inversion and eversion of the calcaneus can be achieved through external and internal rotation of the tibia, respectively. This motion is directly transferred to the calcaneus as the talus is firmly located in the ankle mortise [26] and movement of the talus is further limited by ligaments [27]. Therefore, movement of the subtalar joint may not have an effect on the orientation of the talocrural joint angle. It is also possible that the amount of inversion/eversion applied in this study was not sufficient to alter the AT moment arm. The foot was positioned in 10° of inversion and eversion, respectively. This amount of rotation was selected as it has been reported to be within the range of motion of every person [28,29]. It is not known, however, whether the amount of inversion/eversion at the calcaneus was indeed 10°. It is reasonable to assume that this was not the case due to the deformation of the calcaneal fat pad. In addition to the exact amount of inversion/eversion being unknown, one must also consider that the entire foot was rotated in the frontal plane along its long axis. The subtalar joint axis, however, is oriented at an angle to the frontal and transverse plane [21,27,30] so that an inversion rotation of the foot along the subtalar joint axis is also accompanied by plantarflexion and adduction, while an eversion rotation is accompanied by dorsiflexion and abduction [14]. Therefore, rotation of the foot along the subtalar joint axis rather than the long axis of the foot may have an effect on the AT moment arm. Indeed, the AT moment of most participants in this study did not differ substantially between the three foot positions but three participants showed considerable differences of around 10 mm between foot positions, which would be practically meaningful. It can be speculated that this may be due to the orientation of the subtalar joint axis. In particular, in feet with an increased inclination angle of the subtalar joint axis (angle between the transverse plane and the subtalar joint axis) inversion and eversion would be accompanied by a greater amount of plantarflexion and dorsiflexion, respectively.

A limitation of the COR method is its two-dimensional approach. The assumption is made that the talocrural joint axis is located perpendicular to the sagittal plane. Previous studies, however, show a deviation from the sagittal plane of varying magnitude [9,10,23,31] which can introduce a calculation error. Maganaris *et al*. make the suggestion to multiply the moment arm determined with the COR method with the cosine of the average deviation angle of the talocrural joint axis to arrive at the actual moment arm [32]. While this calculation indicated that the COR method overestimates the AT moment arm, it does not seem suitable given the large range of deviation angles reported for the talocrural joint axis [15,23,10]. Recently, methods to determine the AT moment arm in three dimensions based on magnetic resonance or computed tomography imaging have been developed and these studies show that AT moment arms within the same dataset were smaller when calculated with this (three-dimensional) approach compared to a two-dimensional approach [9,33]. With the foot perpendicular to the lower leg, however, the magnitudes of the three-dimensional AT moment arm reported cluster around the same value of about 50 mm as for previously reported two-dimensional values [9,11,30]. Therefore, the COR method can be considered a fast and valid imaging-based method, but AT moment arms reported in this study are likely to be overestimated due to the two-dimensional nature of the method compared to estimations with a three-dimensional approach.

## 5. Conclusion

In conclusion, this study showed that there is no difference in the moment arm of the AT in different inversion/eversion positions of the foot in healthy, non-pathological adults when estimated with the two-dimensional COR method. The force transmission capabilities of the AT during plantarflexion may, therefore, not be affected by inversion/eversion of the foot and it may not be necessary to take into account a change in the AT moment arm when investigating mechanical properties of the AT during plantarflexion movements in various inversion/eversion positions of the foot in this population. However, results may differ for children or clinical patient groups such as patients with cerebral palsy or osteoarthritis due to altered geometries of the bones of the rearfoot.

## Data accessibility

The datasets from this study are publically available on the Dryad Digital Repository (https://doi.org/10.5061/dryad.3t251) [34].
